# The ENGAGE study: a 3-arm randomized hybrid type 1 effectiveness and implementation study of an in-home, collaborative PCP model of remote telegenetic services to increase uptake of cancer genetic services in childhood cancer survivors

**DOI:** 10.1186/s12913-024-10586-z

**Published:** 2024-02-28

**Authors:** Tara O. Henderson, Mary Ashley Allen, Rajia Mim, Brian Egleston, Linda Fleisher, Elena Elkin, Kevin Oeffinger, Kevin Krull, Demetrios Ofidis, Briana Mcleod, Hannah Griffin, Elizabeth Wood, Cara Cacioppo, Michelle Weinberg, Sarah Brown, Sarah Howe, Aaron McDonald, Chris Vukadinovich, Shani Alston, Dayton Rinehart, Gregory T. Armstrong, Angela R. Bradbury

**Affiliations:** 1 Department of Pediatrics, The University of Chicago, Chicago, IL USA; 2 Abramson Cancer Center and Division of Hematology-Oncology, The University of Pennsylvania, Philadelphia, PA USA; 3 Fox Chase Cancer Center, Philadelphia, PA USA; 4 Columbia University, New York, NY USA; 5 Duke University, Durham, NC USA; 6 Department of Epidemiology and Cancer Control, St. Jude Children’s Research Hospital, Memphis, TN USA; 7 Department of Medical Ethics and Health Policy, The University of Pennsylvania, Philadelphia, PA USA

**Keywords:** Genetic services, Telegenetic services, Remote genetic services, Childhood cancer survivors, In-home, Collaborative PCP model

## Abstract

**Background:**

Germline cancer genetic testing has become a standard evidence-based practice, with established risk reduction and screening guidelines for genetic carriers. Access to genetic services is limited in many places, which leaves many genetic carriers unidentified and at risk for late diagnosis of cancers and poor outcomes. This poses a problem for childhood cancer survivors, as this is a population with an increased risk for subsequent malignant neoplasms (SMN) due to cancer therapy or inherited cancer predisposition. The **ENG**aging and **A**ctivating cancer survivors in **Ge**netic services (ENGAGE) study evaluates the effectiveness of an in-home, collaborative PCP model of remote telegenetic services to increase uptake of cancer genetic testing in childhood cancer survivors compared to usual care options for genetic testing.

**Methods:**

The ENGAGE study is a 3-arm randomized hybrid type 1 effectiveness and implementation study within the Childhood Cancer Survivor Study population which tests a clinical intervention while gathering information on its delivery during the effectiveness trial and its potential for future implementation among 360 participants. Participants are randomized into three arms. Those randomized to Arm A receive genetic services via videoconferencing, those in Arm B receive these services by phone, and those randomized to Arm C will receive usual care services.

**Discussion:**

With many barriers to accessing genetic services, innovative delivery models are needed to address this gap and increase uptake of genetic services. The ENGAGE study evaluates the effectiveness of an adapted model of remote delivery of genetic services to increase the uptake of recommended genetic testing in childhood cancer survivors. This study assesses the uptake in remote genetic services and identify barriers to uptake to inform future recommendations and a theoretically-informed process evaluation which can inform modifications to enhance dissemination beyond this study population and to realize the benefits of precision medicine.

**Trial registration:**

This protocol was registered at clinicaltrials.gov (NCT04455698) on July 2, 2020.

## Background

### Childhood cancer survivors and genetic testing

Germline cancer genetic testing has become a standard evidence-based practice, with established risk reduction and screening guidelines for genetic carriers [1–4]. Yet, many at-risk patients do not have access to genetic services, leaving numerous genetic carriers unidentified and at an increased risk of late diagnosis of cancers and poor outcomes [5–9]. Many areas in the U.S. have limited access to genetic specialists, requiring patients to travel long distances to referral centers to received genetic services. Some patients proceed with testing without a genetic provider (i.e. with their PCP). This has been associated with lower genetic knowledge and satisfaction, and many at-risk patients do not proceed with testing at all [10–13]. Given increasing precision medicine applications and a limited and geographically restricted workforce of genetic providers, innovative delivery models that are responsive to the needs of geographically and sociodemographically diverse patient populations in their local health care systems are needed [8, 14–17].

Suboptimal access to genetic services is an acute problem for survivors of childhood cancer, as many in this population are at high risk for subsequent malignant neoplasms (SMN) because of cancer therapy or an inherited cancer predisposition [18, 19]. A recent study revealed that 12% of survivors had a germline mutation in a cancer susceptibility gene (e.g. *TP53, BRCA1/2*) [20]. Guidelines from National Comprehensive Cancer Network (NCCN) and Children’s Oncology Group (COG) recommend survivors with personal (e.g. sarcoma) and/or family history of cancer be referred for genetic testing to implement appropriate surveillance or preventive measures (e.g. breast cancer surveillance or prophylactic mastectomy in women with *TP53* or *BRCA1/2*) [1, 21]. Yet, less than 15% of survivors have access to genetic services [22]. Further, survivors and their PCPs are largely unaware of survivor’s health risks, and overall adherence to surveillance guidelines is low [23–26]. These data highlight the need for childhood cancer survivors to be referred for genetic counseling and testing, which could reduce morbidity and mortality in this high-risk population.

#### Remote telehealth genetic services

Remotely providing genetic services by phone or videoconference as an alternative to in-person delivery could address access barriers. Randomized studies established that patient-reported outcomes (e.g. knowledge, distress) with remote phone services were no worse than in-person services for cancer genetic testing, although uptake of testing in some studies was lower in the remote phone arm than in-person services [27–30]. Remote videoconference services have been utilized as an alternative to in-person services and have increased during the COVID pandemic [31–36]. In cancer genetics, remote videoconference services have demonstrated feasibility and high patient satisfaction, but published studies are limited, heterogeneous in setting and delivery, nonrandomized, small and have limited patient-reported outcomes [37–42]. Given lower uptake of genetic testing in two studies utilizing phone services, and preliminary data suggesting potential benefits in knowledge with videoconference in community-based populations, further evaluation of the relative benefits of videoconference over phone are needed. Additionally, moderator analyses can help identify who benefits less or more from access to videoconference over phone services [43].

**Fig. 1:**
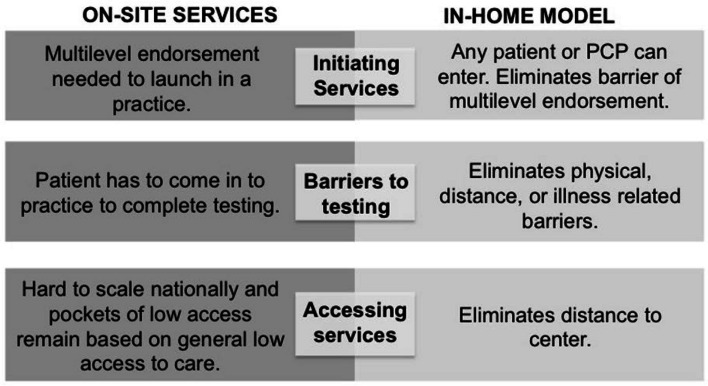
Comparison of on-site to in-home model of genetic services

In a randomized study of remote telegenetic services delivered *on-site at community adult oncology practices,* 80% of patients who met NCCN criteria had genetic services, as compared to 16% (OR 30.5, *p* < 0.001) in the usual care arm (in all cases with their local PCP and not a genetic provider) [44]. While remote genetic services can significantly increase uptake of genetic testing in patients in community practices, the on-site model requires multi-level support within practices and still requires proximity to select community sites, which limits scalability for a nationally distributed population (Fig. 1). Thus, a model where any patient or doctor can access services, where services are provided in the home (eliminating any remaining travel burdens) and are not tied to a participating center, could provide even greater scalability, access to genetic services and uptake of genetic testing, which will be critical to realizing the potential of precision medicine.

#### The in-home, collaborative PCP model for remote genetic services

To address the gap in access to genetic services, we propose to evaluate the effectiveness of an *in-home, collaborative PCP model* of remote delivery of genetic services to increase the uptake of recommended genetic assessment and testing in childhood cancer survivors. A PCP Advisory Board, including 8 Family Physicians, General Internists, and an OB/Gyn that practice in diverse community practices (urban, rural, suburban, Northeast, Southeast, Midwest, South and West Coast), informed the development and procedures of the *in-home collaborative PCP model* and confirm that it is a feasible and acceptable approach (Fig. 1). Advisory board members confirmed the value of access to genetic services, as many PCPs don’t have sufficient expertise to address genetic testing guidelines. They felt many PCPs would be willing to participate if the process was simple and did not require significant provider time. PCPs confirmed that rare and high-risk conditions (e.g. childhood cancer survivors), having patients approach them with requests for services or guideline based recommendations is appropriate (“right place at the right time”). They reported that older PCPs, and those in practices without an electronic medical record or with limited medical and administrative support, might experience barriers to uptake. Recommendations to increase participation included making clear the value of testing, streamlining and simplifying PCP/practice steps, including reminders to minimize burden on the practices, providing access to education materials and answers to frequently asked patient questions and access to the genetic counselor for specific clinical and screening questions even after testing.

### Present study

This study, **ENG**aging and **A**ctivating cancer survivors in **Ge**netic services (ENGAGE), describes a 3-arm randomized Hybrid 1 Effectiveness and Implementation study in a population of 360 Childhood Cancer Survivor Study (CCSS) participants to evaluate the effectiveness of an *in-home collaborative PCP model* of remote telegenetic services to increase uptake of cancer genetic testing in childhood cancer survivors compared to usual care options for genetic testing.

### Objectives

The goal of the ENGAGE study is to evaluate the effectiveness of our *in-home, collaborative PCP model* of remote telegenetic services to increase uptake of cancer genetic testing in childhood cancer survivors compared to usual care options for genetic testing. We hypothesize that this innovative delivery approach has the potential to provide a scalable model that will overcome existing access barriers to services and support optimal patient outcomes in geographically diverse clinical populations, as the indications for genetic testing expand in the era of Precision Medicine.

#### Specific aim 1

Our primary aim is to evaluate the effectiveness of our *in-home collaborative PCP model* of remote telegenetic services to increase uptake of genetic services at 6 months as compared to usual care among childhood cancer survivors who meet criteria for cancer genetic testing. The primary outcome is a composite variable indicating whether a person had pre-test counseling or genetic counseling.

#### Specific aim 2

Our secondary aims are to evaluate the effectiveness of remote videoconferencing to provide greater increase in knowledge and decrease in distress and depression compared with remote phone services (Aim 2a), to examine the moderators of patient outcomes with remote telegenetic services, to understand who benefits less or more from remote services as compared to usual care, and videoconference as compared to phone counseling (Aim 2b), and to estimate intervention costs and incremental cost-effectiveness of the three study arms (Aim 2c).

#### Specific aim 3

Our third aim is to conduct a multi-stakeholder, mixed-methods Consolidated Framework for Implementation Research (CFIR)-informed implementation evaluation to understand patient, provider and system factors acting as barriers or enablers to uptake of counseling and testing in our *in-home, collaborative PCP model* to provide recommendations for future wider implementation of this model to populations beyond the CCSS.

## Methods

### Study design

**Fig. 2:**
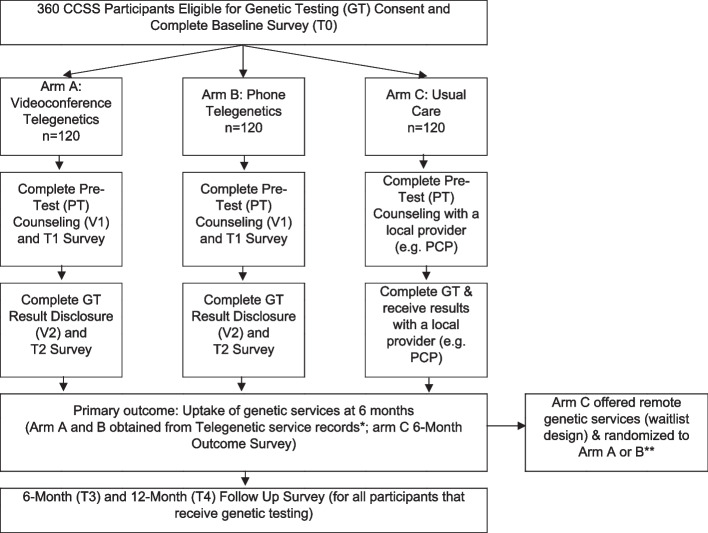
Randomized study schema. *6-Month Outcome Survey also given to participants on Arms A and B that have lost contact with Penn Telegenetics before receiving genetic services; participants in Arms A and B who have not had genetic services can still receive services according to their randomized arm

ENGAGE is a 3-arm randomized Hybrid 1 effectiveness and implementation study, which tests a clinical intervention while gathering information on its delivery during the effectiveness trial and its potential for future implementation. We will randomize 360 childhood cancer survivors (1:1:1), who meet guidelines for germline cancer genetic testing (Fig. 2) to remote in-home telegenetic services by videoconference (Aim A), by telephone (Arm B) or to usual care (Aim C). Equally important, we will conduct a multi-stakeholder mixed-methods CFIR-informed concurrent implementation evaluation to understand patient, provider and system factors associated with uptake of telegenetic services and facilitators and barriers to uptake.

### Setting

**Fig. 3:**
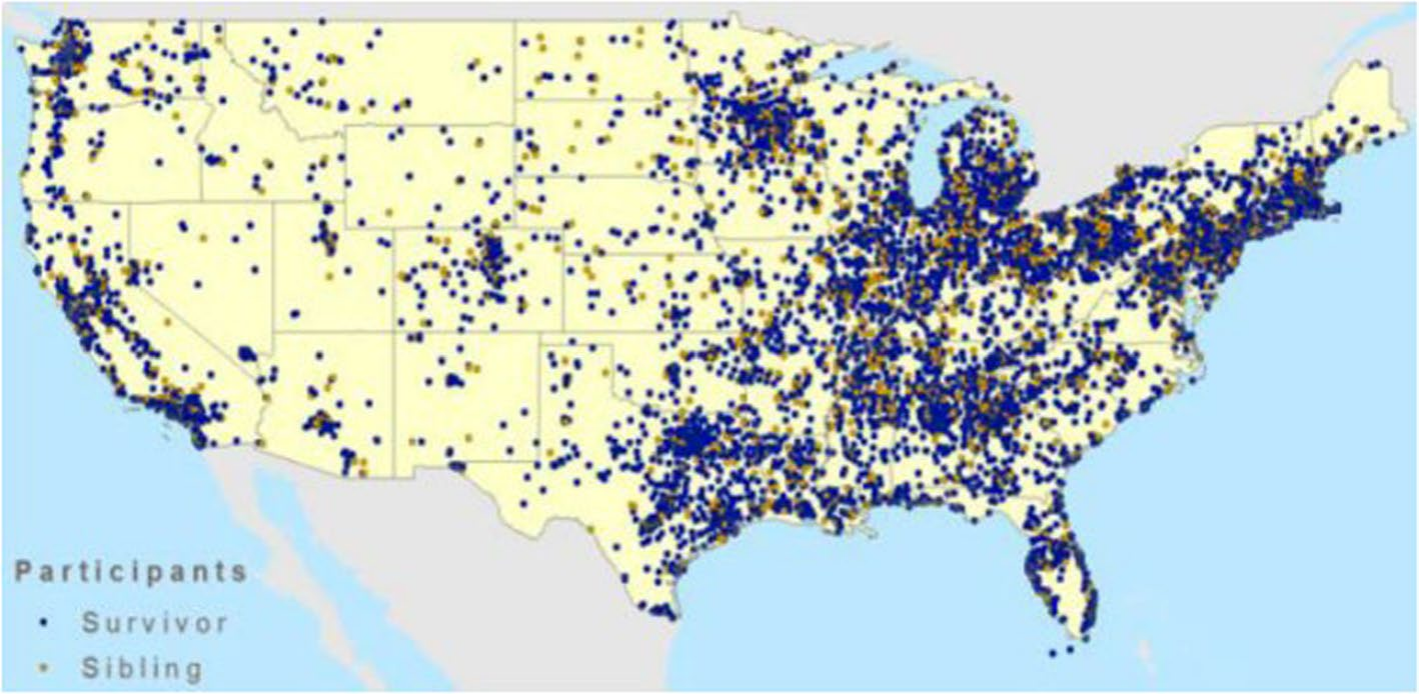
U.S. Distribution of CCSS Participants

This study is being conducted within the Childhood Cancer Survivor Study (CCSS), a multi-institutional North American cohort established in 1994 to evaluate the long-term outcomes of childhood cancer survivors [45]. Including patients diagnosed with their primary cancer from 1970 through 1999, the CCSS now follows the outcomes of 24,735 childhood cancer survivors representing both urban and rural North America (See Fig. 3). Based on data from the Surveillance, Epidemiology, and End Results (SEER) Program, the participants in the CCSS cohort are “similar in terms of gender, race, and cancer type by time interval since diagnosis to those reported in SEER, indicating that the CCSS was representative of the larger U.S. population of childhood cancer survivors” [18]. The health care utilization patterns of the CCSS participants have been evaluated periodically, finding that over 80% of participants (regardless of risk of recurrence or late effects) are no longer followed at their treating cancer center [22, 46–48], and 88% of CCSS participants report that they have an identified PCP.

Remote genetic counseling services in this study are provided through the Penn Telegenetics Program, which was founded in 2012 originating from NCI funded research evaluating remote phone and real-time genetic services in community practices without genetic services [49]. The program has provided remote genetic services by phone or videoconference in cancer and neurogenetics in over 100 national sites through research or clinical contracts [49, 50]. More recently, Penn Telegenetics developed the Patient Access Program (PAP), permitting individual patients and providers across the nation to access Penn Telegenetic services. In the PAP, Penn Telegenetic Counselors collaborate with local health care providers to provide genetic services in the home. This model includes a physician registration process to facilitate this collaborative care model and this model is the basis for the in-home, collaborative PCP model in this study and several other nationally accruing remote services studies (NCT04353973, NCT05427240). At the initiation of ENGAGE, 66 patients (in 17 different states) and 32 physicians had successfully registered with the program and 50 (76%) patients had either completed (*n* = 44) or scheduled (*n* = 6) genetic counseling, providing key preliminary data supporting this model. The Penn Telegenetics genetic counselors (GCs) are licensed in all US States as required by state licensure laws [51].

The ENGAGE study is co-implemented with teams at the University of Pennsylvania (Penn Telegenetics), University of Chicago and St. Jude Children’s Research Hospital (Coordinating Center for the Childhood Cancer Survivor Study). Given that primary intervention activities occur with the Penn Telegenetics Program, the University of Pennsylvania is the IRB of record.

### Study participants

#### Eligibility

Eligibility criteria include individuals enrolled in CCSS, who are 18 years or older, able to understand and communicate in English, reside in the United States, have a history of a CNS tumor or sarcoma (excluding Ewing sarcoma), one or more SMN or have a family history of cancer qualifying them for genetic testing according to the NCCN guidelines [1]. Exclusion criteria include uncorrected or uncompensated speech defects that would lead to the participant being unable to communicate effectively with a medical provider, uncontrolled psychiatric/mental condition or severe physical, neurological, or cognitive deficits rendering the individual unable to understand study goals or tasks. Participants who have already received clinically appropriate multi-gene panel genetic testing are excluded. We elected not to exclude individuals without a usual source of care (e.g. primary care provider) or those without health care insurance. For individuals in these categories, we provide educational materials and resources for obtaining care or insurance.

#### Recruitment

All potential participants are identified through the CCSS Coordinating Center. The CCSS Coordinating Center uses a combination of electronic (email and text message), mailed, and phone-based recruitment methods to contact all potential participants. The introductory letter includes five videos reviewing the value of genetic testing for childhood cancer survivors (brief animated video, physician explaining the benefit of genetic testing, GC explaining the steps of genetic testing, GC explaining costs and addressing genetic discrimination and GC discussing overcoming apprehension regarding genetic testing), including a study invitation letter by email with five reminders and follow up calls.

The ENGAGE study utilizes a participant portal called myLTFU, a HIPAA-compliant interface that allows direct and interactive messaging and web portal-based data collection. This portal allows participants to consent, view educational content, complete surveys, track study progress outcomes, upload and download documents and pictures, and securely interact with the study team. Communication with participants takes place through the DatStat Connect Platform, which allows for pre-programmed messages and workflows to be activated by situational triggers. Recruitment invitations started in August, 2021.

#### Enrollment goals

As outlined below, our enrollment goal for the primary aim is 120 per arm or 360 participants total. We elected to contact childhood cancer survivors of osteosarcoma, soft-tissue sarcoma, and multiple primary cancers, as no additional information is needed to confirm their eligibility for genetic testing. If necessary, we would next contact individuals with a first-degree relative with ovary, male breast or pancreatic cancer as they meet NCCN criteria with this history alone. Next, we would contact those with a family history of colon, uterine or breast cancer, although family history details would need to be assessed to determine if they meet NCCN criteria for genetic testing. As shown in Table 1, we expect to have sufficient CCSS participants to meet our enrollment goal of 360, although if needed we could extend to all childhood cancer survivors and assess family history for eligibility (*N* = 19,154). Importantly, to increase the generalizability of our findings, we seek to enroll 30% of participants from under-represented minorities.

**Table 1:** Characteristics of living CCSS Participants with targeted cancers for initial recruitment (*n* = 7269)

Characteristic	N	%
Current age Median (range)	39.0 (18.0–67.0)	
Years from dx Median (range)	32.0 (17.0–47.0)	
Male Gender	3758	51.7
Race/Ethnicity		
White	6133	84.4
Black	437	6.0
Other/Mixed race	591	8.2
Hispanic	642	9.1
Graduated College	3320	46.0
Insured	6299	87.2
Primary cancer diagnosis		
Osteosarcoma	1175	11.7
Soft-tissue sarcoma	852	16.2
History of an SMN	169	2.3
Family history		
FDR with ovary, male breast, or pancreatic cancer	181	2.5
Family history of breast, colon, or uterine cancer	964	13.1

*FDR* First degree relatives

### Study arms

**Fig. 4:**
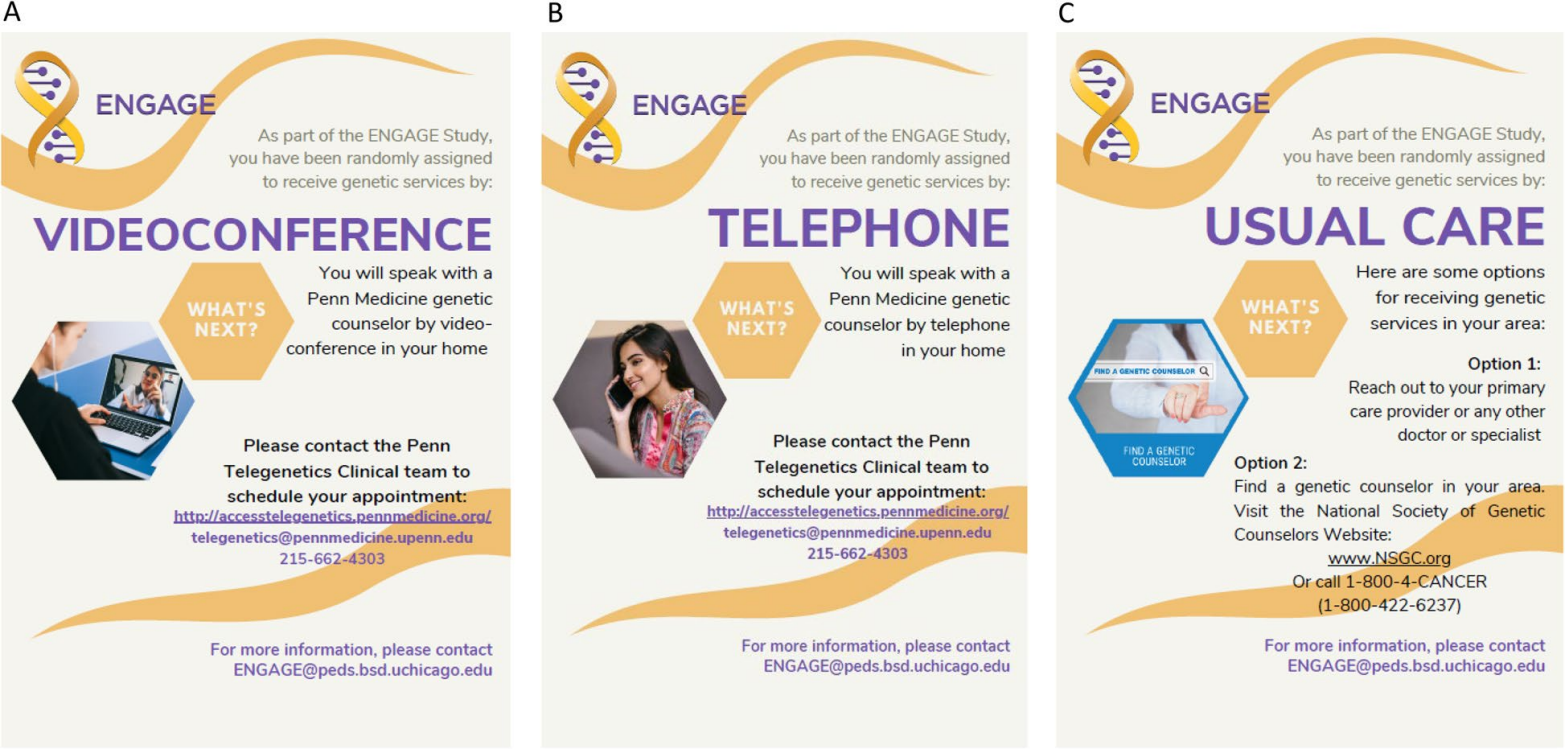
Randomized arm information flyers on how to obtain genetic services

Once participants have completed informed consent and the baseline (T0) survey, they are randomized into one of the potential arms of the study. Randomization assignments are determined by a permuted block design and stratified by gender (e.g. male, female, other). After randomization, participants are sent a flyer via the myLTFU portal that have instructions for contacting the Penn Telegenetics team to schedule an appointment (for Arms A and B) or usual care options (Arm C) for obtaining genetic services [e.g. ask PCP or providers, use the National Society of Genetic Counselors website (www.nsgc.org)] (Fig. 4). In all arms, participants need to take the first step to initiate services, using information provided in the randomization flyer.

#### ARM A: Remote telegenetic services by videoconference

Participants who contact Penn Telegenetics in Arm A will complete their pre-test counseling session by videoconference in the home or their selected personal location with a Penn Telegenetic Counselor (GC) utilizing communication protocols from related studies [44, 52]. The Telegenetics team will contact the participant up to 6 times (3 calls and 3 emails) to schedule their pre-test counseling session. If a scheduled participant does not show for their appointment, they are contacted 3 times to reschedule. Participants are provided links to download secure videoconferencing software on their home computer or device. The Penn Telegenetics team utilizes a HIPAA-compliant technology platform (Blue Jeans) for videoconference visits. In our communication protocols, if videoconference technology fails, GCs will convert the session to phone. Based on our experience at study start (> 800 telegenetic sessions across studies and clinical contracts), failures occur only 4% of the time, although this may be higher when adapting our model for in-home videoconferencing.

Consistent with standard clinical practice, GCs will review personal and family history (FH), the risks, benefits and limitations of genetic testing, testing options based on their personal and family history (e.g. TP53 testing or a panel of cancer susceptibility genes) and the costs associated with genetic testing. The Penn GC works with the patient to determine appropriate testing and which lab to utilize for testing, provides instructions for completing the test kit and reviews information on testing costs. GCs order the testing through clinical labs (e.g. Invitae or Ambry Genetics) and the labs send test kits to the participant’s home. Through the ENGAGE portal, participants get 3 email and 3 text reminders to return their test kits.

After results are available, the Penn GC shares results with the patient by videoconference at a scheduled visit, and provides the PCP with the chart note, summarizing the results, implications, cancer risk estimates, and standard risk-reducing or screening strategies and implications for relatives. This chart note is also provided to patients, consistent with routine clinical care. Penn GCs are available to answer PCP questions and facilitate referral to regional centers for genetic carriers as indicated. Patients are recommended to follow-up with their PCP to implement any screening and medical (including risk reduction) recommendations.

#### ARM B: Remote telegenetic services by phone

Participants who contact Penn Telegenetics in Arm B complete their pre-test counseling session by phone in the home or their selected personal location with a Penn Telegenetic Counselor (GC) utilizing communication protocols from related studies [30, 44, 52]. The remainder of procedures for pre-test counseling, genetic testing and post-test counseling sessions are as described above for Arm A.

#### ARM C: Usual care

As above, participants in the usual care arm will receive print materials reviewing ways to obtain genetic services. These include speaking with their doctor about testing or accessing the National Society of Genetic Counselors, “find a counselor” function on their website. At 6 months participants will be contacted to evaluate if they had genetic testing (see 6-month status survey in Effectiveness Outcomes below). All Arm C participants who have not had genetic testing at 6 months will be offered remote telegenetic services in a waitlist design (Fig. 2). If they accept remote services, they are re-randomized to Arm A or Arm B. This future potential for Telegenetic services (e.g. waitlist design) is not shared with them at the time of enrollment. Rather, they are told that after they complete the 6-month status survey.

#### Genetic testing

Genetic testing in all arms will be clinical cancer genetic testing through standard clinical commercial labs, consistent with real-world practice and as indicated based on their personal and family history. Genetic testing is intentionally not covered by the study to reflect “real-world” practice. For the subset of participants who are not able to get testing covered by insurance, commercial labs offer financial assistance programs and out-of-pocket testing is often no more than $250. Based on prior experience in populations meeting NCCN criteria for testing, we anticipate this will be < 5% of participants [44].

#### Genetic testing and PCP collaboration (Arms A and B)

If patients elect to proceed with genetic testing, they will be required to provide their PCP’s contact information, who will be the ordering provider as a licensed physician is required to order genetic testing in most states. Consistent with the Penn Telegenetics Patient Access Program procedures, the Penn Telegenetics Team contacts the PCP office, provides information on Penn Telegenetics Program and requests that the provider register with our program. Registration includes providing their credentials and contact information, so that the GC can collaborate with the PCP to facilitate testing. PCP practice staff will be permitted to complete registration on behalf of the PCP. As above, these procedures have been successful to date in a pilot study and were informed by a PCP Advisory Board.

### Outcomes

#### Conceptual model (Fig. 5)

**Fig. 5:**
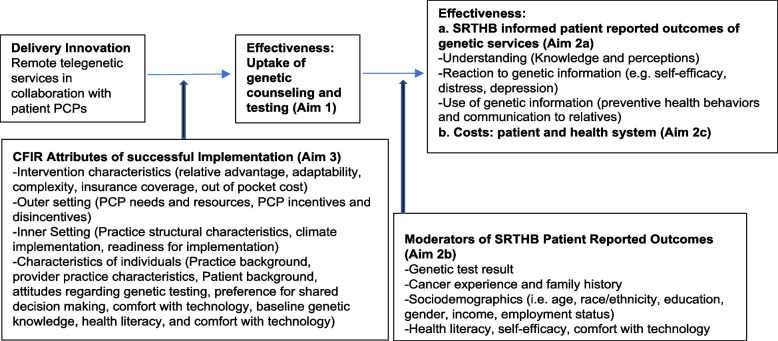
Conceptual model to evaluate outcomes of innovations to delivery of genetic services

Outcomes in the ENGAGE study have been informed by our conceptual model grounded in The Self-Regulation Theory of Health Behavior (SRTHB) to evaluate innovations in the delivery of genetic services [49, 52–54]. This model proposes that the reaction to, and use of health (genetic) information is the product of an individual’s understanding, knowledge, and perception of the disease threat and risk reduction behaviors [53, 55–61]. It emphasizes “common-sense” representations rather than medical or scientific definitions, and incorporates individual cognitive, emotional, familial, and cultural experiences that might contribute to individual variability in understanding. Thus, it has been proposed that the SRTHB is an ideal framework for considering the outcomes of genetic testing [54, 59, 62]. Understanding the moderators of outcomes with delivery innovations are critical to understanding who benefits more, or less from delivery innovations. The literature supports the hypothesis that cognitive, psychological, and behavioral factors will be moderated by biological test results, [63–66] cancer history, [64, 67–69] sociodemographic factors (e.g. education, race/ethnicity) [64, 69–71] and cognitive and emotional factors (e.g. health literacy [72–74] and self-efficacy [75, 76]).

Our conceptual model is also guided by the Consolidated Framework for Implementation Research (CFIR), which provides and overarching theoretical framework to evaluate barriers, enhancements, and adaptations to increase successful implementation [77]. Even after health-related interventions have proven efficacy and effectiveness, many fail to translate into clinical settings [78, 79]. Thus, there is increasing recognition of the importance of evaluating and addressing barriers to implementation across diverse health care settings. The Consolidated Framework for Implementation Research (CFIR) provides an overarching theoretical framework to evaluate barriers, enhancements and adaptations to increase successful implementation [77]. Based on our stakeholder interviews and implementation science experts, we have included selected constructs from each of the 5 major CFIR domains (intervention characteristics, outer setting, inner setting, characteristics of individuals and process) as they relate to our intervention (Fig. 5) which will be evaluated in our multi-stakeholder mixed-methods process evaluation (Aim 3). As in other studies, [80–82] evaluation of CFIR components is expected to identify patient, provider and system barriers and enablers of our remote delivery interventions and increase future dissemination to increase access and adoption of genetic services.

#### Effectiveness outcomes (Aim 1)

The primary outcome of Aim 1 is a composite variable of whether the participant had genetic testing or counseling by six months, and uptake of genetic counseling and identification of genetic carriers are considered secondary outcomes. Uptake of counseling, testing and identification of carriers at 6 months (Aim 1) will be obtained through study records for Arms A and B (intervention arms) and through the 6-month status survey in Arm C (usual care) and participants in Arms A and B who did not complete remote services. This time frame was selected as it allows for a range of delays in completion of counseling and testing and was found to be a reasonable time frame in our prior study [44]. The 6-month status survey includes two closed ended questions for the primary outcome (“Have you completed genetic counseling/testing in the past six months?)”. Secondary closed and open-ended items evaluate how they obtained usual care genetic services (provider, setting, method, and costs), their experience (e.g. what was easy/hard? do you have questions about your results?). For those who did not complete genetic services, secondary items explore barriers to services and what might change their interest in genetic services. The 6-month status survey is designed to be interviewer administered, but if necessary can be completed self-administered. Participants who complete the 6-month outcome survey receive a $75 gift card code.

#### Effectiveness outcomes (Aim 2)

Outcomes to evaluate the effectiveness of remote videoconference as compared to phone services are shown in Table 2. Upon completion of the T0 survey, T1 survey, and T2 survey, participants receive a $25 gift card code. Participants who complete the T3 survey and T4 survey receive a $30 gift card code.

1. *Understanding of genetic information* will be assessed at baseline (T0, all Arms), post genetic pre-test counseling session (T1), post genetic test (T2), 6 months post genetic testing (T3), and 12 months post genetic testing (T4) for those in Telegenetic Arms A and B. *Knowledge of genetic disease* will be evaluated using The KnowGene Scale, a 16-item scale administered to patients after genetic testing and/or genetic counseling to measure their understanding of the health implications of genetic testing results. It includes health implications to oneself as well as relatives. This measure covers penetrance, actionability, limitations of current technology, and monogenic inheritance patterns [83].
*Perception of genetic disease* will include three items (T0-T4), utilized in related research and evaluating perceived risk of developing a second cancer on a verbal scale and perceived numerical risk, as well as a single item evaluating perceived timeline [56, 59].
2. *Reactions to genetic information* will be assessed at baseline (T0, all Arms), post genetic pre-test counseling session (T1), post genetic test (T2), 6 months post genetic testing (T3), and 12 months post genetic testing (T4) for those in Telegenetic Arms A and B).
  - *General anxiety and Depression* will be assessed by the 4-item each short Patient Reported Outcomes Measurement Information System (PROMIS), a system of highly reliable, precise measures of patient-reported health status for physical, mental, and social well-being [84–87].
  - *Disease-specific distress* will be measured using the 8 –item Impact of Events Scale (IES) [88, 89], also with strong internal consistency (alpha = 0.82–0.90) in genetic delivery studies [90–92].
  - *Satisfaction with genetic services* will be assessed with 9-items evaluating satisfaction with genetic services (T1 and T2). These items have been utilized in our related studies evaluating alternative and traditional genetic delivery models (alpha = 0.73–0.85) [90–96].
  - *Satisfaction with telemedicine* will be assessed with 8-items adapted for genetic counseling sessions and utilized in our preliminary studies [44, 97].
  - *Multidimensional responses to genetic testing*, including positive responses and uncertainty will be assessed using the Multi-dimensional Impact of Cancer Risk Assessment Questionnaire (MICRA) at T2-T4. The MICRA is a 21-items scale that has been utilized in many genetic studies to evaluate distress, uncertainty and positive responses to receipt of genetic test results [98]. The final item was excluded as it is not included in the three subscales and assessed regret which will be assessed in a separate scale.
  - *Decisional regret* (T2-T4) will be assessed with the 5-item validated Decision Regret Scale used frequently in related genetic studies [99, 100].
3. *Use of genetic information* will include endorsement of behavior items utilized in the CCSS and related studies (T0, T3, T4). Behaviors will include cancer specific (screening, prophylactic surgery, chemoprevention) and general risk modifying behaviors (e.g. diet, exercise, tobacco and alcohol use). Communication with providers and relatives following disclosure will be measured as well.
4. *Cost of remote services and usual care*: Estimation of intervention costs will adopt a societal perspective, including relevant direct medical and nonmedical costs borne by providers, payers and patients. Information to estimate intervention costs will be collected from study billing and payment records (e.g. print materials, postage) and telegenetic staff logs (personnel time). Intervention cost estimates will not include the cost of resources used solely for research purposes. Information about patient time and travel and other out-of-pocket costs (e.g. co- payments), will be obtained through the participant surveys. Service use will be assessed at T1, T2, T3 and T4 timepoints.
5. *Moderators of patient outcomes* with remote telegenetic services (Aim 2b) will be collected at T0 only and include:
  - *Sociodemographic data* will include race/ethnicity, education, marital status, gender, age, employment status, household income, health insurance (yes/no) and a usual source of medical care (yes/no) which will be collected in the Baseline Survey (T0).
  - *History of cancer* will be collected in the Baseline Survey (T0).
  - *Genetic test result* (positive, negative, true negative and test/genes included) will be obtained from Telegenetic Service records and the 6-Month Outcome Survey.
  - *Health literacy* will be assessed at baseline (T0) with 3 health literacy Brief Literacy Screen (BHLS) screening items which have been validated to detect inadequate health literacy in clinical medical populations [101].
  - *Computer literacy* will be assessed at baseline (T0) with selected items from the NCI Health Information National Trends Survey (HINTS), including internet and social media use (8 items), electronic medical record use and perceptions of privacy (14 items) [102].
  - *Self-Efficacy* will be measured at baseline (T0) with the 4-item PROMIS Self-efficacy short form for managing chronic conditions, which has been validated in adults with medical conditions (including cancer) and has good internal consistency (alpha = 0.85–0.92) [103].
  - *Financial wellness* will be measure at baseline (T0) with 2 items from the Personal Financial Wellness Scale [104].
  - *Psychological impacts due to COVID-19* will be assessed using 15–16 items previously utilized in the CCSS to assess the impact of the COVID-19 pandemic on CCSs. The final item asking about number of individuals in the household is excluded at T2. While this protocol is independent of COVID-19, there is potential for COVID-19 events and impact on psychosocial outcomes to impact other primary and secondary outcomes. The measure is included to allow for analyses to evaluate the impact of these on study outcomes [105].

**Table 2:** Effectiveness outcomes and measures

CONSTRUCT	T0	T1	T2-T4
**Moderators of patient outcomes**			
Sociodemographics	X		
Cancer history	X		
Family history	X		
Health literacy	X		
Comfort with technology	X		
Self-efficacy	X		
COVID Impact	X		X
**Outcomes**			
***Uptake counsling, testing, identification carriers at 6 months*** ^a^ (Telegenetics records for Arms A/B & the 6-Month Status Survey in Arm C)			
***Understanding of Genetic Information*** ^b^			
Test result recall			X
Knowledge of genetic disease	X	X	X
Perceived risk	X	X	X
***Reactions to genetic information*** ^b^			
Anxiety and Depression	X	X	X
Cancer specific distress	X	X	X
Satisfaction with genetic services and telemedicine		X	X
***Behavioral use of genetic information*** ^b^			
Performance of behaviors	X		X
Cost (patient and system)^b^		X	X

T0 = baseline (within 30 days of visit 1, can accept within 45 days. beyond that it needs to be updated), waitlist usual care particiants will need to complete a new baseline, T0b; T1 = survey post pre-test counseling, Visit 1 (ideally collect 1–3 days and up to 7 days. accept up to 14 days but from 7–14 days we may elect to drop and impute). T2 = survey immediately post genetic testing disclosure (same timing as T1); T3/T4 survey = 6/12-month post genetic testing disclosure (idealy get within 14 days of 6/12 month mark but accept within 60 days of 6 month or 12 month mark)

^a^Aim 1

^b^Aim 2

#### Implementation outcomes

Implementation outcomes are determined by identifying key CFIR-informed constructs across the five domains to evaluate factors related to uptake of remote telegenetic services, implementation facilitators, and barriers and adaptations (Fig. 5, Table 3). Key informant interviews will be conducted with patients, PCPs and practice staff as part of our Aim 3 implementation evaluation. Key informants will include:

1. Patients: A purposive sample of 20–40 patients in intervention arms A and B (e.g. up to 20 from each arm). Throughout recruitment, diversity variables will be tracked in order to inform ongoing sampling and maximize representativeness in terms of gender, race/ethnicity, education and uptake (enrolled but did not complete counseling, completed counseling and completed testing);
2. PCPs: 15–30 PCPs identified by participants to collaborate for delivery of remote telegenetic services in Arms A or B. As above, we will seek to maximize representativeness in terms of gender, race/ethnicity, years in practice, type of practice and uptake (registered with patient and completed services, registered but did not complete services, declined registration);
3. Medical office staff: 15–30 office staff affiliated with PCPs will be contacted. Staff can participate even if the PCP declines participation in the process evaluation.

We seek to continue recruitment to the above enrollment goals or until data saturation occurs across the theoretical domain (e.g. no new themes introduced within the constructs included in key informant interviews) [106]. Interviews will be conducted by phone by research staff, audio recorded and transcribed.

**Table 3:** CFIR constructs, measures, samples items and sources

Measures	Sample Items	PCP Reg	Patient Survey	TG service records	Key Informant Interviews^a^
**Intervention Characteristics**					
Relative advantage	Perception that remote services provides an advantage over existing care options		X		X
Adaptability	Perception that the intervention could be modified to meet practice, provider or patient needs, recommendations for improvement		X		X
Complexity	Perception that services were too complex		X	X	X
Cost	Perception that remote services genetic counseling or testing would be too costly for patient, practice (cost or resources)		X	X	X
**Outer setting**					
Provider needs and resources	Awareness of genetic risk and cancer prevention; importance within practice				X
Provider incentives and disincentives	External mandates for genetic testing (e.g. accreditation)				X
**Inner Setting**					
Structural characteristics	EMR, medical staff #, structure			X	X
Climate for implementation	Previous innovation implementation, compatibility with practice, relative priority, quality metrics/rewards				X
Readiness for Implementation	Available staff resources, practice experience with genetic services			X	X
**Characteristics of Individuals**					
Practice background	Type of practice, practice setting	X			
Provider practice characteristics	Years in practice, gender, comfort with genetic testing, champion for genetic testing				X
Patient background	Age, race, ethnicity, education, insurance		X		
Patient attitude toward genetic testing	Attitudes about genetic testing scale (8 items)^3^		X		
Patient preference for shared medical decision making	Control Preference Scale (5 items)^4^		X		
Patient comfort with technology	HINTS 5, Cycle 1 selected items related to internet and social media use (8 items) and electronic medical record use and perceptions of privacy (14 items)^5^		X		
Patients baseline genetic knowledge, health literacy and affect	See Table 2 and corresponding measures in Section C6. Effectiveness outcomes		X		
**Process**					
Planning	Planning process for PCPs, quality of materials to introduce the program, steps for PCPs/practice and supports provided		–	X^b^	X
Opinion leaders	Who in the practice was most influential, how did they influence others or what could have helped them to be more effective				X
Champions	Who in the practice helped ensure that all steps were completed? What did they do to make the practice successful?			X	X
Reflecting and evaluation	What procedures are working? Which are not? What can we change to make the process easier? etc			X^c^	X^c^

*PCP Reg* PCP Registration, *TG* Telegenetics

^a^Key informant interviews will include patients (*n* = 20–40); PCPs (15–30) and PCP office staff (15–30)

^b^includes notes from our PCP Advisory Board planning meetings

^c^includes regular personal and team debriefing about what is working and not working about the process of collaborating with PCPs and their practices to ensure implementation of remote genetic services

### Data analysis plan

#### Primary effectiveness analysis: remote telegenetic services compared to usual care (Aim 1)

We hypothesize that participants who are randomized to remote telegenetic services will have significantly higher uptake of genetic testing or genetic counseling at 6 months. We will use Fisher’s Exact tests to compare uptake of genetic testing and pre-test counseling between the arms, and these will be two separate binary 0/1 variables. The primary comparison groups will be the usual care and wait list group versus the combined remote telegenetics groups (videoconference plus phone) prior to randomization of the waitlist group. For the primary analyses, we will use an intention to treat approach where comparisons are made between randomization arms. In secondary analyses, we will compare the randomization arms to investigate if potential confounders (e.g. age) are balanced among the randomization arms, and this will be done via pairwise Wilcoxen-rank sum tests and Chi-squared tests as appropriate. If any potential confounders are not found to be balanced among the randomization arms (i.e. *p* < 0.10 for any potential confounders in pairwise comparisons), we will use multiple logistic regressions of uptake to investigate the randomization arm effect. In these models, we will include randomization arms as binary (0/1) indicator covariates (leaving one out as the reference), and we will also include the potential confounders as covariates in the regressions.

We do not expect substantial missing data, although there may be loss to follow-up over time. For the primary outcomes, we will assume that those lost to follow-up did not have pre-test counseling or testing (i.e. failures under the intention-to-treat paradigm). Data from our community practice stakeholder interviews suggests this is reasonable since many patients do not get genetic services even when referred. In secondary analyses, we will account for missing data using the multiple imputation technique of Raghunathan with 25 imputed datasets [107]. We will contrast the results obtained through imputation and those obtained from complete case analyses to study if missing data bias could be substantially affecting our inferences.

#### Sample size justification for aim 1

We chose our sample size to have sufficient power for both Aims 1 and 2. For Aim 1, the sample size was selected to detect average differences in the primary endpoint (a composite variable indicating either genetic testing or genetic counseling) between the primary randomization arms of interest. The primary comparison groups will be the usual care/waitlist group versus the combined remote telegenetics groups (phone or video conference as one group); the primary analysis will consider the groups prior to the waitlist rerandomization. Preliminary data demonstrated that uptake of genetic testing from an ongoing registered randomized trial of remote telegenetic services vs. usual care for cancer genetic testing in community practices was at least 53% (29/55 with uptake) in the intervention arm, but only 17% (4/24 with uptake) in the control arm. With 120 patients in the usual care/waitlist group and 240 patients in the combined telegenetics groups with complete data (after accounting for loss to follow up), we will have > 99% power to detect a similar difference in uptake of genetic testing. This assumes a 1% Type I error rate (2-sided) and the use of Fisher's Exact Test. We set the Type I error rate to a conservative 1% to partially account for multiple hypothesis testing when including secondary outcomes and potential moderators in Aim 3. Given the magnitude of the pilot data differences, we anticipate excellent power for the moderator/subgroup analyses. The comparison of the usual care/waitlist group to the combined telegenetics group will be the comparison used to determine if the study accomplishes its primary objective. The uptake of pre-test counseling (Aim 1 secondary objective) and comparison between the phone and videoconference groups (see Aim 2a) are secondary objectives.

#### Secondary effectiveness analysis: remote videoconference compared to phone services (Aim 2a)

We hypothesize that remote telegenetic services by videoconferencing will be associated with greater decreases in cancer related distress and depression and increases in knowledge when compared to phone services. We will compare the usual care at baseline group to the combined arms receiving remote services at baseline. For comparisons between the remote videoconferencing versus phone groups, we will assign participants to the remote group assigned at baseline, or after rerandomization for the waitlist group. Our primary change scores for the first three variables will be change between baseline and immediately post genetic testing. For the waitlist group, there will be a second baseline measurement after the usual care period has ended. For the 3 primary analyses in Aim 2, we will use T-tests. We will assess balance of potential confounders (e.g. age, race, study site, waitlist assignment) between arms using T-tests or Fisher’s exact tests as appropriate. Waitlist group will be considered a confounder in these secondary regression analyses. We will use multiple linear regression models to control for potential confounders inadequately balanced. We will include in the models the confounding variables and indicator (binary 0/1 variables) to indicate randomization arms (leaving one group out as the reference). For longitudinal analyses, we will examine time trajectories using regressions estimated by Generalized Estimating Equations (GEE) to account for within subject temporal correlation. Panel time will be included via indicator variables. We will also include interaction terms between randomization arm and time indicators to investigate temporal effects [108]. We will repeat analyses, but assign participants to per-protocol (i.e. restricting sample to those who properly used the assigned method of disclosure) or as-treated groups (e.g. assigning all participants to a group based on the method of disclosure used, regardless of assignment). For Aim 2 secondary outcomes, we will use multiple linear regressions for continuous outcomes and multiple logistic regressions for binary outcomes using GEE-estimation as described.

#### Sample size justification for aim 2a

We further chose our sample size such that we would have sufficient power for Aim 2 comparisons of the videoconference versus phone arms. After re-randomization of the waitlist group, we anticipate potentially having 180 people/arm randomized to the videoconference or phone arms. Based on prior experience, we anticipate that loss to follow-up will be less than 28% in each arm, leaving us with 130 evaluable participants with follow-up data in each group (180–180*28% = approximately 130). Some of the loss to follow-up will occur prior to the waitlist group being rerandomized. We used our pilot data to determine power for this aim (see Table 2 for estimates). We assumed 85% power and a 1.67% Type I error rate. We used a 1.67% Type 1 error rate (2-sided) by applying a Bonferroni correction for the three outcomes of interest to the typical 5% Type I error rate (5%/3 = 1.67%). We see in Table 4 that we have excellent power for all three arms with at least 95/arm, which is well below our expected 130/arm anticipated sample size. Table 4 also demonstrates that we have sufficient power for subgroup analyses, with a sample size of just 22/arm needed for knowledge comparisons between arms.

**Table 4:** Sample size calculations for Aim 2a (comparison of phone versus videoconference services)

Variable	Change scores^a^ (SD) of phone vs. videoconferencing	Number needed^b^
Knowledge	+ 5.7 (11.2) vs. + 18.6 (12.6)	22/arm
Cancer specific distress^c^	+ 3.6 (12.4) v. -2.6 (12.3)	95/arm
Depression	-0.2 (2.0) vs. -1.6 (2.0)	50/arm

^a^Baseline to post genetic testing

^b^85% Power, 1.67% alpha

^c^intrusive subscale

#### Moderator analysis (Aim 2b)

To evaluate moderators of patient cognitive, affective and behavioral outcomes (Aim 2b), we will use logistic regressions in which we include variables of interest as covariates. For moderation (i.e. effect modifier analyses), we will include indicator variables for randomization arms in the GEE-estimated multiple linear (for continuous outcomes) or logistic regressions (for uptake). We will also include panel time indicators, the potential moderator variables, and all two-way and three-way interactions among the arm/time/moderator variables. Interactions are created by multiplying two variables together [108]. We will examine non-time moderators separately, and will examine multiple regression models with potential confounding variables.

We will repeat the analyses but assign participants to per-protocol (i.e. restricting sample to those who properly used the method of disclosure as assigned) or as-treated groups (e.g. assigning all participants to a group based on the method of disclosure used, regardless of group assignment).

#### Intervention costs and incremental cost-effectiveness analysis (Aim 2c)

The economic impact of remote telegenetic services (Aim 2c) will be assessed by performing: 1) cost analysis and 2) cost-effectiveness analysis. The goal of the cost analysis is to estimate the cost of delivering remote telegenetic services, by phone and videoconference, compared to usual care. As above, the cost analysis will take a societal perspective, including the costs of all resources consumed for the implementation and delivery of remote genetic services. Separately, we will examine costs from the health system perspective that can inform decisions about the provision and reimbursement of services, and ultimately will influence the effective dissemination and implementation of remote telegenetic services beyond the study. Cost-effectiveness will be estimated as the incremental cost per (1) additional survivor who receives genetic testing, and (2) additional mutations detected. The numerator of the incremental cost-effectiveness ratio (ICER) will include intervention costs and patient time and travel costs, estimated as described above. In the first analysis, the denominator will be defined by the primary trial endpoint of genetic testing received within 6 months. Because receipt of genetic testing is the measure of effectiveness in this case (i.e. the denominator of the ICER), neither the cost of testing nor the cost of related services provided after genetic testing will be included in the numerator. For the second analysis, the numerator of the ICER will include both genetic test costs and any related health care service costs (additional tests, visits, counseling) associated with genetic testing at 6 months. Incremental cost effectiveness will be estimated using standard methods, and sensitivity analysis will be used to assess the impact of assumptions and uncertainty on results and conclusions [109, 110].

#### Mixed-methods CFIR-informed implementation analysis (Aim 3)

We hypothesize that our CFIR-informed multi-stakeholder mixed-methods process evaluation will identify barriers to uptake and inform recommendations for future adaptation and sustainability*.* We will quantitatively characterize factors associated with uptake using T-tests (continuous predictors), chi- squared tests (categorical predictors), and multiple logistic regressions (multivariable models). We will control for randomization arm in multivariable models. Qualitative data will be analyzed using a deductive content analysis approach and CFIR as the coding framework [77, 82, 111]. The qualitative data will include patient surveys and key informant interviews, notes from the PCP Advisory Board and notes and debriefing from the telegenetics service and research team meetings. Guided by consensual qualitative methods [112, 113], two independent coders review notes and assign responses to CFIR constructs. Finally, using a convergent mixed-methods approach [114], we will merge the quantitative and qualitative data, organized by the 5 CFIR domains to evaluate which constructs were associated with, or more prevalent among: a) patients who had testing and those who did not and b) PCPs who registered and their patient completed testing, PCPs who registered and their patient did not complete testing, PCPs who never registered (were contacted by the patient but did not support testing through our model of remote telegenetic services). As in other studies [80–82], evaluation of CFIR components is expected to identify patient, provider and system barriers and enablers of our remote delivery interventions and increase future dissemination to increase access and adoption of genetic services.

## Discussion

We hypothesize that our adapted in-home, collaborative PCP model of remote telegenetic services has the potential to provide a scalable model that will overcome existing access barriers to genetic services and support optimal patient outcomes in geographically diverse clinical populations, as the indications for genetic testing expand in the era of Precision Medicine. Further, we believe the CCSS provides an ideal “real-world’ population of socio-demographically and geographically diverse patients to empirically evaluate this novel delivery model.

### Limitations and potential challenges

There are potential limitations we may encounter in this study. We are relying on the 6-month status survey to evaluate what occurs in the usual care arm. Loss to attrition for any of the surveys may result in some missing data. We plan to monitor completion and consider reducing survey burdens or increasing incentives to obtain generalizable data. While we have had strong PCP willingness to register with the Penn Telegenetics Program, we may find some PCP are not willing to collaborate to facilitate genetic services for patients in the intervention arms. Understanding these barriers, if they occur, could inform future interventions or modifications to optimize access to genetic services in the primary care setting. Some patients may not have coverage for testing or face high out-of-pocket costs. While we expect this to be limited, understanding real-world cost barriers to testing will be helpful to advancing equitable access to precision medicine.

### Potential for impact and implications

The ENGAGE Study will provide critical empiric data on the effectiveness of an in-home, collaborative PCP telegenetic service model to increase the uptake of genetic testing and a theoretically-informed process evaluation which can inform modifications to enhance dissemination beyond this study population and to realize the benefits of precision medicine.

## Data Availability

No datasets were generated or analysed during the current study.

## Abbreviations

AYA: Adolescent and young adult
CCSS: Childhood Cancer Survivor Study
COG: Children’s Oncology Group
CFIR: Consolidated Framework for Implementation Research
ENGAGE: Engaging and Activating Cancer Survivors in Genetic Services
GC: Genetic counselor
HINTS: Health Information National Trends Survey
NCCN: National Comprehensive Cancer Network
PCP: Primary care physician
SMN: Subsequent malignant neoplasms
SRTHB: Self-Regulation Theory of Health Behavior
